# GPs' reasons for referral of patients with chest pain: a qualitative study

**DOI:** 10.1186/1471-2296-10-55

**Published:** 2009-07-31

**Authors:** Rudi Bruyninckx, Ann Van den Bruel, Karin Hannes, Frank Buntinx, Bert Aertgeerts

**Affiliations:** 1 Department of General Practice, Katholieke Universiteit Leuven, Leuven, Belgium; 2 Research Institute Caphri, Universiteit Maastricht, Maastricht, The Netherlands

## Abstract

**Background:**

Prompt diagnosis of an acute coronary syndrome is very important and urgent referral to a hospital is imperative because fast treatment can be life-saving and increase the patient's life expectancy and quality of life. The aim of our study was to identify GPs' reasons for referring or not referring patients presenting with chest pain.

**Methods:**

In a semi-structured interview, 21 GPs were asked to describe why they do or do not refer a patient presenting with chest pain. Interviews were taped, transcribed and qualitatively analysed.

**Results:**

Histories of 21 patients were studied. Six were not referred, seven were referred to a cardiologist and eight to the emergency department. GPs' reasons for referral were background knowledge about the patient, patient's age and cost-benefit estimation, the perception of a negative attitude from the medical rescue team, recent patient contact with a cardiologist without detection of a coronary disease and the actual presentation of signs and symptoms, gut feeling, clinical examination and ECG results.

**Conclusion:**

This study suggests that GPs believe they do not exclusively use the 'classical' signs and symptoms in their decision-making process for patients presenting with chest pain. Background knowledge about the patient, GPs' personal ideas and gut feeling are also important.

## Background

Chest pain can be a sign of an ischemic or non-ischemic cardiac disease, a gastro-oesophageal or pulmonary disease, a musculoskeletal disorder or psychiatric illnesses, all of which require specific treatment [1-14]. For patients with an acute coronary syndrome, urgent referral to a hospital is imperative because mortality decreases if thrombolysis or PTCA can be carried out quickly [10].

Severe prolonged chest pain of acute onset accompanied by other specific symptoms is rarely a decision-making problem. Attacks of chest pain that are experienced by patients as not very severe and prolonged, but distressing enough for them to contact a general practitioner, present a more difficult problem in diagnosis and management [15].

In a diagnostic meta-analysis, we were not able to define an important role for individual signs and symptoms in the diagnosis of acute myocardial infarction or acute coronary syndrome (ACS), except chest wall tenderness on palpation, which largely rules out those diseases in low-prevalence settings [11].

This is confirmed by Abu Hani et al. who found that GPs use criteria not present in classical textbooks when diagnosing acute coronary syndrome (ACS), such as person-specific discrepancies between previous and actual consultations.

Pauker and Kassirer defined a threshold as the disease probability at which no action changes into action (starting medication, performing medical imaging or blood tests, reassuring the patient), based on the balance between risks and benefits of acting versus not acting. Similarly, in patients with chest pain, the GP has to decide on referral of the patient rather than making a specific diagnosis of ACS [16,17]. In fact, the diagnosing labelling 'does my patient have an acute coronary syndrome?', is less important than the action of referral because chest pain patients with lung embolism or aneurysm should be referred as well.

However, little is known about the grounds on which GPs decide to refer a patient with chest pain. The aim of our study was therefore to identify GPs' reasons for referring or not referring patients presenting with chest pain.

## Methods

We invited 85 GPs in the first author's region (Vilvoorde, Belgium) by invitation during a local CME meeting, by personal letter two months later, and by phone-call after another two months as a reminder, to participate in an interview-based study exploring why some patients presenting with chest pain were referred and others were not. To increase the number of participants, we also sent an email to 320 GPs in the region between Brussels and Antwerp. The GPs were asked to call us immediately after seeing a patient presenting with chest pain, regardless of their initial diagnosis and regardless as to whether or not the patient was referred. Cases were not limited to patients with an acute coronary syndrome, but the GPs were actively encouraged to include any patient consulting with chest pain.

All interviews were carried out within at most two days after the contact, usually at the GP's office. The semi-structured interview consisted of three main questions: how does the GP act in general when seeing a patient with chest pain and what is considered important when referring or not referring a patient; how would the GP describe this specific patient and what actions were or were not undertaken for which reasons; and how does the GP cope when confronted when making an incorrect decision concerning a patient with chest pain. A more detailed list is given in Table 1. However, the semi-structured nature of the interview meant that the interviewer consistently encouraged the GP to focus and go into more detail. The list of questions was only there to prevent omissions in the topics to be addressed. For each result reported, we provide a textual transcription, enabling the reader to judge whether the statement is sufficiently correct and specific. All the interviews were led by the first author (RB). The interviews were transcribed verbatim and entered into ATLAS.ti 5.0 software to assist data analysis [18]. Before analysis of the interviews, an initial codebook, containing four themes, based on the experience as GP, was developed: history taking, physical examination, patient-related reasons, GP-related reasons. Then the lead researcher undertook an inductive stepwise approach to data analysis. All issues of interest were marked in the data, and labeled and compared with other interview excerpts for similarities and differences. The second researcher independently analyzed all the interviews based on this codebook, also marking and labeling the issues of interest. During the analysis the codebook was adapted with new themes to better classify themes that emerged. All interviews were analyzed again using this final codebook by those two researchers. Conflicts were solved by discussion between two researchers. Data collection was stopped when saturation was achieved, defined as the identification of no new codes in the last two interviews. The themes were classified by the first author (RB) as 'specific for ACS' or 'not specific for ACS' and also marked in the results section.

**Table 1:** Structure of the interview.

1	When you see a patient presenting with chest pain, what do you do?
2	What did you do with this specific patient?
3	Was there something special?
4	Was there something that scared you?
5	Were there alarm signs or symptoms?
6	Were there other arguments that influenced your decision?
7	Were there also non-medical arguments influencing your decision?
8	Were there also arguments contradictory to your decision?
9	There are different ways of referring. Sending patients to the emergency department, yes or no. Ambulance yes or no, emergency rescue team, yes or no. Could you explain your decision.
10	This is a neutral question! What arguments do you use for referring patients to the cardiologist or emergency department, for calling an ambulance or for calling the emergency rescue team?
11	Were you satisfied with your decision?
12	In the past, had you already made a mistake concerning a patient with chest pain?
13	Was there an emotional influence?
14	Did this change your attitude?
15	What is your opinion of this interview?
16	Would you like to add other questions?
17	Were there any questions you disliked?

The study was approved by the Medical Ethics Committee/Clinical Research of the Catholic University of Leuven. (ML 2378)

## Results

Saturation was reached after 21 interviews with 21 GPs; GP characteristics are described in Table 2. Seventeen GPs were from the local group (response rate: 20%) and four responded to the email (response rate: 1%). Two interviews were badly recorded and not usable. Two GPs reported on two patients each, thus the interviews reported on 21 patients of 19 GPs.

**Table 2:** GP characteristics.

Gender	
Male	18
Female	3
Age	43 (26–52)^a^
GP in practice	16 (1–27)^a^
Practice location	
Urban	10
Rural	11
Practice organization	
Group	3
Duo	6
Solo	9
Trainee	3
Twenty-one GPs interviewed in total	

^a ^Mean followed by the range in brackets

Fourteen of these patients were female (mean age 52). Six were not referred, seven were referred to a cardiologist (one of them refused) and eight were referred to the emergency department (one of them refused). Of those seven who accepted referral to the emergency department, three were transported by relatives, two by their GP and two by ambulance.[Table 3]

**Table 3:** Patients' characteristics.

*P Nr*	*Age*	*Gender*	*History*	*Action*
1	31	M	Precordial pain, for 4 weeks already, no risk factors	Cardiologist
2	70	F	Retrosternal pain, also shoulder and back, since the morning, cholesterol, diabetic.	ED, transport by family
3	67	M	Oppressive pain, sweating, since the morning, prostate metastasis, ST-elevation	ED, transport by GP
4	53	M	Oppressive pain, sweating, for minutes, dyspnoea, PTCA	ED, transport by ERT
5	75	F	Sometimes oppressive pain, for 10 days already, bypass, diabetes	Cardiologist
6	30	M	Oppressive pain, stress, for weeks, no risk factors	No referral
7	82	F	Epigastric pain, nightly, for 3 days already. Cholesterol, ECG normal	ED, transport by family
8	67	F	Angor precordial oppression, for months already, stress	Cardiologist
9	80	F	Angor precordial oppression, since?, diabetes, cholesterol	Cardiologist
10	50	F	Precordial oppression, tachypnoea, for 2 days already, familial problems	ED, refused
11	65	F	Precordial pain, 1 week, angor-like, stress	No referral
12	75	F	Precordial pain, since?, hypertension	Cardiologist
13	50	F	Precordial pain, rhythm disorder, one week	Cardiologist
14	43	F	Precordial pain, also back, since the previous night, ECG ST depletion	ED, transport by GP
15	30	F	Precordial pain, extrasystole, weeks?, stress	No referral
16	80	M	Retrosternal pain, hours, CABG antecedents, ECG ST elevation	Transport by ambulance
17	67	F	Retrosternal pain, only at night, for a week, cholesterol, obesity, diabetes	No referral
18	30	M	Pain hemithorax, no risk factors	No referral
19	82	F	Retrosternal pain, hours, CABG antecedents, ECG ST elevation	ED, transport by ambulance
20	17	F	Retrosternal pain, one day, fever	No referral
21	62	M	Retrosternal pain, for 10 days already, CABG	Cardiologist, refused

M = male, F = female, ED = emergency department, ERT = emergency rescue team

All the GPs interviewed gave very personal accounts of their reasoning. All the GPs stated the importance of history taking.

-And then I've actually already got an idea of what it is, before I investigate further, I'm 95% certain what it is. (GP17)

The decision reasons mentioned by GPs could be divided into three general categories:

the GP's background knowledge about the patient, independent of the current episode; the current clinical presentation; and the GP's personal ideas.

### Background knowledge of the patient

#### Risk factors for ACS

[specific ACS] Previous coronary events and coronary risk factors were always considered important factors in the referral decision. However, two GPs mentioned that the presence of risk factors is not necessarily indicate ACS.

-and given the medical history, i.e. two bypass operations, diabetes and high blood pressure, I had reason enough to think that there was something wrong with his heart again. (GP5)

-..I've had patients like that in my office, people who have no risk factors at all and then have a heart attack. But that's extremely bad luck, unlike an obese diabetic who hasn't looked after himself for 20 years, who is a very high risk..(GP17)

### Differences in the patient's behavior

[not specific ACS] Discrepancies between previous and actual consultations were often stated as a trigger for the diagnosis of heart disease and a reason for referring.

-She's normally an active woman, her house is always well cared for but that day she hadn't done a thing the whole day! So that influenced my decision. (GP2)

#### Patient tends to play down symptoms

[not specific ACS] The belief that the patient tends to play down the seriousness of his complaints was also an important factor in the decision-making process. It made the GP more suspicious about the possibility of a serious disease needing referral.

-a complainer, but if there's really something physically wrong, she's so good at pretending there isn't a problem. (GP8)

### Current clinical presentation

#### Specific pain

[specific ACS] Pain on exertion, radiating pain, oppression-like pain were of major importance for the decision. Retrosternal pain was the key for referral. Other localizations were less important.

-I almost always ask if it feels tight and then I demonstrate it with my hand, like the tightening of a screwdriver, or pressure of the foot on the ribcage because I usually find that specific enough. (GP5)

-I think that if the pain is not retrosternal, for example if it is lateral, there's a lot less chance of heart problems. (GP15)

#### Time factor

[specific ACS] The start, frequency and duration of the pain are used to decide on referral. Pain of longer duration, constant or very frequent pain dissuaded GPs from referring.

-If it lasts longer, one hour or longer or even half a day, then I find that less alarming; it's more likely to be the result of stress or something...if it is only 5 minutes, then I'm much more likely to suspect angina than if it lasts two hours. (GP20)

-Something that occurs very frequently, several times a day without being too much of a problem, means it's a lot less acute in my book. (GP7)

#### Clinical examination

[specific and not specific ACS] In general, GPs perform the clinical examination to rule in some diseases such as rhythm disorders, lung diseases and gastro-intestinal diseases. Chest wall tenderness is mentioned to rule in musculoskeletal diseases. But in the end, the clinical examination is not considered very influential as regards the referral decision.

-Just blood pressure, and doing an auscultation as well to exclude heart arrhythmias, because you never know. A palpation of the abdomen. An auscultation of the lungs, it's rare to get thoracic pain of the lungs, so it would have to be pneumonia that causes the pain. (GP5)

#### ECG

[specific ACS] When a GP decides to perform an ECG, it is mainly used to rule in acute coronary syndrome. In cases of an abnormal result, this was an important reason to refer. Sometimes, the combination of a normal ECG, the absence of risk factors and the presence of pain of longer duration was used to rule out ACS.

-At that moment I was pretty sure that it was not an acute heart attack because I had an ECG of someone at rest, who had been complaining for a few days; I would have seen something at that moment if it had really been a heart attack. (GP5)

#### The typical coronary heart disease patient

[specific ACS] This picture was used in a positive and a negative way. If the picture was positive, urgent referral by ambulance was performed.

-It actually depends a little on how the patient looks. If they are pale and sweaty, and really don't look well, then I will always call the medical rescue team. (GP2)

-A young man with pain in the left hemithorax, frequent and daily shooting pain when at rest, not sharp, just a few seconds. He's not able to move during those moments and he is anxious. The clinical examination was completely normal. No ECG was taken; it's probably something musculoskeletal or perhaps nothing, just anxiety. (GP18)

#### Gut feeling

[not specific ACS] Without the 'typical coronary heart disease patient' picture, the appearance of the patient – their looks – was often stated as very important in the decision-making process. It is more than the combination of the signs and symptoms: it is more like a 'gut feeling'.

-She came in and she was different from usual: she looked drawn. It was really striking: her countenance was so sharp. (GP2)

-Basically, if it looks fishy, I refer them immediately. (GP1)

### GP's personal ideas

#### Uncertainty or explicit certainty

[not specific ACS] Uncertainty as well as explicit certainty about an acute coronary syndrome were reasons for referring the patient.

-If I'm not sure and anxious about it, then I refer. (GP4)

-But I think that if I'm reasonably sure it's a heart attack, I will always call for the medical rescue team. (GP4)

#### Age and cost-benefit

[not specific ACS] Younger patients were referred more readily to the emergency department than older ones. Older people were sometimes not referred because the expected benefit of the referral was considered to be limited.

-For the whole of society too; why should society pay so much money if you know that the prognosis is very limited. Incidentally she died a few days later. (GP17)

#### Perception by the GP of a negative attitude from the medical rescue team towards GPs

[not specific ACS] Some GPs objected to the attitude of these personnel, which made it more difficult to refer.

-The whole scene; the sirens and waking up all the neighbours, and those men, my room is too small for them with their 5 cases and oxygen and you stand there...And when they remember to think of it, they ask if you've given the patient anything. (GP18)

#### Recent contact of the patient with a cardiologist

[specific ACS] If the patient has been given the all-clear by a cardiologist, can give the GP a false feeling of certainty about the absence of an acute coronary syndrome and influence his referral attitude.

-I have to say that I hesitated a little, especially because he had been to the cardiologist a few days before and had been told everything was alright. (GP14)

#### Errors in the past

[specific and not specific ACS] Ten GPs mentioned an error relating to chest pain patients in the past. The reasons for the error were not recognizing ACS because of resemblance to gastric problems or chest wall tenderness on palpation; or because of the patient's behavior, e.g. the patient is always consulting with minor complaints or always complaining; and for GP-related reasons, e.g. the GP waited too long before making a home visit and the patient was transported by ambulance without the assistance of a medical rescue team. This error created various feelings such as regret and the realisation of the huge responsibility. Talking to the family is reported as being important, but the memory of the error keeps some GPs awake at night. Some GPs mentioned that an error influences subsequent decisions, in that they are more careful and their threshold for action is lower.

-Then I can't go straight to sleep when I come home. I continue to think about it for a long time. (GP10)

-You feel bad in a way because you made a wrong diagnosis, but I don't lie awake at night; this sort of thing happens, and then it's a case of not justifying yourself to these people, but instead having a chat with them about it. (GP21)

-I have to be honest, it sometimes makes me a little over cautious as well. Then I may be too quick to refer a patient so as to be sure nothing is missed. (GP17)

## Discussion

### Summary of the main findings

This study suggests that the background knowledge on the patient, the patient's current clinical presentation and the GP's personal opinions are used by GPs when deciding on whether or not to refer a patient with chest pain. *Background knowledge *on the patient – coronary risk factors, differences in behavior, playing down the seriousness – was an important factor in the decision-making process about whether or not to refer. For those factors, knowing the patient is essential.

The *current clinical presentation*: clinical examination in particular is used to rule in diseases other than acute coronary syndrome which need no referral. An ECG was used to confirm the presence of an acute coronary syndrome and refer the patient. A normal ECG was a reason for not referring, but only in combination with a long duration of pain and the absence of risk factors. A gut feeling is sometimes more important than the presence of individual signs and symptoms.

The *GP's personal ideas *– the patient's age, perception of a negative attitude from the medical rescue team, recent patient contact with a cardiologist, past errors – were factors in the decision-making process. Sometimes, uncertainty about the diagnosis causes an unnecessary referral. Referring older people has a higher threshold than referring younger people because of the expected smaller benefit.

### Strengths and weaknesses of the study

The interviews were taken very shortly after the GPs had seen a patient with chest pain. This is important, as the GPs may reinterpret their diagnostic reasoning in the light of information from a cardiologist or based on the evolution of the patient's condition.

All the interviews were carried out by the principal researcher, himself a GP experienced in medical research and qualitative studies. Being a 'man of the field' and knowing the reality of the situation, certainly had an impact on the interviews, the participants and the analysis. The data were analysed by the principal researcher, who developed the initial codebook, and independently by a second researcher. The second researcher was a sociologist, who introduced a broader, non-medical perspective to the study topic.

The recruitment of GPs who were willing to participate in the interviews was a difficult process. The prospect of being judged and facing possible criticism may have been a reason for non-collaboration.

Loss of time – without financial compensation – could be another reason. More reminders may have been necessary. Although e-mail is an easy way to recruit GPs, the response is limited. On the other hand, the quality of the interviews of the GPs recruited by e-mail was very high.

Compared to the general population of GPs in Flanders, the participating GPs were similar in age and practice organization – single-handed or group practice – but not in gender: female GPs are underrepresented in our sample. Our data did not reveal any difference in reasoning between the three females and the three trainees, and the male group of participants. Of course, gender bias is always possible. The same applies – although in reverse – for the patient population: women are overrepresented here. But female patients with chest pain may present a more diagnostic and decision-related dilemma, in which the selection of the sample does not necessarily threaten the validity of the results. In addition, in qualitative studies, the goal is not to recruit a representative sample of participants to quantify opinions, but rather to elicit all possible opinions and views on a specific subject. In our data, saturation was reached, which suggests that all important criteria were identified.

In spite of the recruitment difficulties, all the interviews were conducted with highly motivated GPs. The GPs responded honestly and voluntarily to the interviewer. Although what doctors say they do is not the same as what they actually do, we believe the quality of the interviews was high [19]. The latter was illustrated by the ten GPs explaining cases where they possibly made an error.

### Previous studies

Abu Hani et al. identified the importance of differences in pain characteristics and the 'typical coronary heart disease patient', the patient's behaviour, the presence of standard cardiovascular risk factors and a tendency to play down the seriousness of the complaints by the patient [20]. They were the first to explore the kind of background knowledge that GPs used in their clinical decision-making process when diagnosing possible coronary heart disease. They also described the importance of pain on exertion, the time factor and their combination. In our study these criteria were also found to be influential in the decision to refer patients with chest pain.

Others have found that the 'typical' symptoms of myocardial ischemia are well known by patients [21]. This 'typical' presentation creates a potentially dangerous expectation among patients that chest pain or discomfort should be present before calling emergency medical services [22].

The importance of 'certainty' and 'gut feeling' for GPs when referring patients with chest pain has already been demonstrated by Buntinx et al. [12,13].

We have shown in another quantitative study that, in the case of diagnostic uncertainty, 26% of the patients presenting with chest pain were urgently referred to the emergency department and 53% were not urgently referred to the specialist [23]. In this study we found that about 60% of patients with chest pain were not referred, suggesting a selection of minor or less 'typical' cases.

The importance of the GP's gut feeling was also described by Van den Bruel et al. in her GP-based study on diagnosing serious infections in children [24].

Our criterion that the 'perception of a negative attitude from the medical rescue team' increases the referral threshold is in line with Tod's finding, when it was demonstrated that the referral threshold decreases when the consultant was easily approachable and communicated well with the patients and the GPs [25].

### Future research

The new reasons for referral mentioned in our study should now be further evaluated for their effect in a subsequent quantitative study, in a synthesis of qualitative studies or both. Hopefully, these studies will further enhance our understanding of the referral decisions made by GPs for patients with chest pain.

## Conclusion

This study suggests that GPs believe they do not exclusively use the 'classical' signs and symptoms in their decision-making process for patients presenting with chest pain. Background knowledge about the patients, GPs' personal ideas and gut feeling are also important. 

What is already known on this subject

- In general practice the low prevalence, the early and often diffuse stages of coronary heart disease are factors making this diagnosis difficult.

- Discrepancies between previous and actual consultations alert the GPs to coronary heart diseases.

- Based on the threshold theory of Pauker and Kassirer, the GP has to decide whether or not to refer a patient consulting with chest pain.

What this study adds

Reasons for referral of patients presenting with chest pain were the GP's background knowledge on the patient, the patient's clinical presentation and the GP's personal opinions and ideas. In particular, a change in behaviour, typical presentation, a GP's gut feeling, and the perception of a negative attitude from the medical rescue team influence a GP's referral decision. Clinical examination is used to exclude and an ECG to include the possibility of an acute coronary syndrome.

## Competing interests

The authors declare that they have no competing interests.

## Authors' contributions

RB, AV, FB and BA designed the study. RB conducted the interviews. RB and EA analyzed the data. RB wrote the first draft of the article. AV, FB, KH and BA provided substantial subsequent contributions. BA supervised the study design and analysis. All authors read and approved the final manuscript. RB is guarantor.

## Pre-publication history

The pre-publication history for this paper can be accessed here:


